# Is the Macronutrient Intake of Formula-Fed Infants Greater Than Breast-Fed Infants in Early Infancy?

**DOI:** 10.1155/2012/891201

**Published:** 2012-09-27

**Authors:** Shelly N. Hester, Deborah S. Hustead, Amy D. Mackey, Atul Singhal, Barbara J. Marriage

**Affiliations:** ^1^Abbott Nutrition, Champaign-Urbana, IL 61820, USA; ^2^Abbott Nutrition, 3300 Stelzer Road, Columbus, OH 43219, USA; ^3^Institute of Child Health, University College London, London WC1N 1EH, UK

## Abstract

Faster weight gain early in infancy may contribute to a greater risk of later obesity in formula-fed compared to breast-fed infants. One potential explanation for the difference in weight gain is higher macronutrient intake in formula-fed infants during the first weeks of life. A systematic review was conducted using Medline to assess the macronutrient and energy content plus volume of intake in breast-fed and formula-fed infants in early infancy. All studies from healthy, term, singleton infants reporting values for the composition of breast milk during the first month of life were included. The energy content of colostrum (mean, SEM: 53.6 ± 2.5 kcal/100 mL), transitional milk (57.7 ± 4.2 kcal/100 mL), and mature milk (65.2 ± 1.1 kcal/100 mL) was lower than conventional infant formula (67 kcal/100 mL) on all days analyzed. The protein concentration of colostrum (2.5 ± 0.2 g/100 mL) and transitional milk (1.7 ± 0.1 g/100 mL) was higher than formula (1.4 g/100 mL), while the protein content of mature milk (1.3 ± 0.1 g/100 mL) was slightly lower. Formula-fed infants consume a higher volume and more energy dense milk in early life leading to faster growth which could potentially program a greater risk of long-term obesity.

## 1. Introduction

Breastfeeding has a number of short- and long-term benefits for health, but the underlying mechanisms for its effects on long-term outcomes are uncertain. One of the most widely cited advantages of breastfeeding is a lower risk of long-term obesity [1–4] and cardiovascular disease [5–7], but whether these effects are due to sociobiological differences between infants breast- or formula-fed or to the nutritional composition and intake of breast milk remains controversial. Several mechanisms for the long-term advantages of breastfeeding have been proposed, but recently we suggested that these effects were a consequence of slower early growth in breast-fed compared to formula-fed infants—the growth acceleration hypothesis [6].

More than twenty-five studies now support the hypothesis that faster weight gain (upward centile crossing for weight) in infancy influences, or programs, a greater risk of long-term obesity [8, 9] and cardiovascular disease [6, 8–10]. This association has been seen for obesity in adults and children, in high-income and low-income countries [8–10] and is consistent for cohorts over the last 80 years [8]. The “critical window” for the effects of growth is not known, but slower weight gain in the first few weeks (regardless of gestation or birth weight) is associated with a lower risk of later obesity [11, 12], insulin resistance [13], endothelial dysfunction [5], and adult obesity [14]. Therefore, differences in weight gain between formula- and breast-fed infants in the first postnatal weeks, when breast-fed babies often lose weight compared to weight gain in babies given formula [15, 16], could partially explain long-term programming advantages of breastfeeding [6].

The difference in early weight gain between formula-fed and breast-fed infants is likely to be related to differences between formula and breast milk in both the composition and volume of intake of colostrum (days 1–5) and transitional breast milk (days 6–14). In fact, in contrast to the composition of human milk which varies with the age of the infant, formula composition is constant and designed to meet the nutrient requirements for the whole of the first six to 12 months of life. However, while there has been extensive research on the nutritional composition of human milk, relatively few studies have focused on the composition and volume of intake during the first few weeks after birth. We therefore conducted a systematic review of the literature and meta-analysis of available data on the macronutrient content of human milk and the volume of milk intake in breast-fed and formula-fed infants in the first weeks of life. Such data could help our understanding of the possible mechanisms by which breastfeeding benefits long-term health and helps in the primary prevention of obesity and cardiovascular disease, and in the development of preventative strategies for obesity in formula-fed infants.

## 2. Methods 

### 2.1. Search Strategy

A literature search investigating the macronutrient and energy content of human milk and volume of milk intake in breast-fed and formula-fed infants in the first two weeks of life was conducted using the National Library of Medicine (MEDLINE). Additional studies were identified from a hand-search of relevant journals and a search of references from identified studies. Details of the search strategy used are shown in Table 1. Since most published studies were observational, a meta-analysis was conducted as described by Stroup et al. [17]. This method was used previously to investigate metabolizable energy (ME) intake in older breast-fed infants [39].

All studies that reported breast milk energy and macronutrient concentration during the first month of life from mothers who were exclusively breastfeeding healthy, term, singleton infants were included. Only volumes of milk intake for either exclusively breast-fed or formula-fed infants were included in the analysis, only studies which reported original data were eligible, and duplicate publications (e.g., in reviews) were excluded. Studies only reporting graphical data were excluded, since estimating values from graphs would increase error in the meta-analysis. Energy and macronutrient values of breast milk were arbitrarily divided into three categories; colostrum (1–5 days), transitional milk (6–14 days), and mature milk (>14 days).

Studies were excluded if they used methods to analyze the macronutrient and energy content of breast milk previously shown to be invalid. For example, studies using nonspecific methods to analyze carbohydrates were not used since they may overestimate lactose concentration, compared to lactose-specific methods [40]. Also, since breast milk energy content varies due to both diurnal variation and variation between fore milk and hind milk [31], only studies that used acceptable sampling methods (e.g., collecting a complete feed, a mid feed, or at the beginning and end of a feed) were included [19].

Estimates of breast milk intake expressed as g/day were converted to mL/day using a correction for the density of human milk of 1.031 g/mL [49]. The meta-analysis values for gross energy content of breast milk were converted into metabolizable energy (ME) levels by multiplying the classic Atwater factors 4, 4, 9 kcal/g for carbohydrate, protein, lipid, respectively, and by assuming 93% of gross breast milk energy reported by bomb calorimetry methods is metabolizable [39]. The conversion of breast milk gross energy values to metabolizable energy allows for a more direct comparison of formula and breast milk energy as labeling of infant formulas utilizes metabolizable energy.

Since triacylglycerols account for 98% of the lipids, data from studies reporting total triacylglycerols concentrations were analyzed as total lipid content [50]. The carbohydrate content of breast milk was taken as the total lactose content, since lactose is the main carbohydrate in breast milk. The oligosaccharides and other sugars were not included in the estimation of carbohydrate in human milk. The calculation of protein included nonprotein nitrogen which includes components such as free amino acids, urea, uric acid, and nucleotides as these components make up 20 to 25% of the total nitrogen in milk [51]. 

Comprehensive Meta-Analysis, version 2 (Biostat, Inc., NJ, USA), was used to perform a meta-analysis of the macronutrient and energy content of breast milk and volume intake of both formula and breast milk by infants during the first weeks of life. Random effects models were used to calculate summary means and SEM for each of the parameters. All data reported from the individual studies are expressed as mean ± SEM.

## 3. Results

### 3.1. Macronutrients in Breast Milk 

#### 3.1.1. Lipids

Our literature search identified 25 potentially eligible papers. After screening these for eligibility, 20 studies were included with a total of 390, 257, and 567 breast milk samples available for analysis of colostrum, transitional milk, and mature milk, respectively. The mean lipid content of breast milk increased from 2.2 ± 0.2 g/100 mL (range 1.0 to 3.0 g/100 mL) in colostrum, to 3.0 ± 0.1 g/100 mL (range 2.5 to 3.8 g/100 mL) in transitional milk, and 3.8 ± 0.1 g/100 mL (range 2.8 to 4.9 g/100 mL) in mature milk (Table 2). Lipid concentrations of both colostrum and transitional milk were lower than that commonly found in formula (3.7 g/100 mL) but similar to lipid concentrations in mature milk. 

#### 3.1.2. Carbohydrates

Our literature search identified 30 potentially eligible papers. We identified 21 eligible studies with 265, 337, and 476 breast milk samples for analysis of colostrum, transitional milk, and mature milk, respectively. Average carbohydrate content of breast milk increased from 5.6 ± 0.6 g/100 mL (range 2.6 to 7.6 g/100 mL) in colostrum to 5.9 ± 0.4 g/100 mL (4.1 to 6.8 g/100 mL) in transitional milk and to 6.7 ± 0.2 g/100 mL (5.0 to 8.3 g/100 mL) in mature milk (Table 3). The carbohydrate content of human milk at all stages of lactation was less than that of formula (commonly 7.6 g/100 mL). 

#### 3.1.3. Protein

We identified 29 potentially eligible papers, and 21 were included with a total of 433, 308, and 415 breast milk samples available for colostrum, transitional milk, and mature milk, respectively. Mean milk protein concentration declined with duration of lactation (Table 4) from 2.5 ± 0.2 g/100 mL (range 1.4 to 6.5 g/100 mL) in colostrum to 1.7 ± 0.1 g/100 mL (1.3 to 2.5 g/100 mL) in transitional milk and 1.3 ± 0.1 g/100 mL (0.8 to 2.1 g/100 mL) in mature milk. Protein concentration in colostrum and transitional milk was greater than commonly found in formula (1.4 g/100 mL), while the protein content of mature milk was slightly lower. 

### 3.2. Intake of Breast Milk and Formula

Our literature search identified 31 potentially eligible papers for breast milk intake. We screened these for eligibility, and 25 were included with a total of 148, 123, 109, 109, 139, 132, and 407 intake values for day 1, day 2, day 3, day 4, day 5, day 7, and >14 days, respectively. We identified 12 potentially eligible papers for infant formula intake, and 9 were included with a total of 157, 157, 129, 128, 128, 126, 188, and 136 formula intake values available for day 1, day 2, day 3, day 4, day 5, day 6, day 7, and >14 days, respectively. To ensure that values for breastmilk and formula intake were comparable between the two groups and to focus on early feeding, the upper age limit was established at 6 weeks. The range of intake was from 15 days to 6 weeks with the majority of the data at 1 month of age.

Most studies measured the total volume of breast milk intake by weighing the infant before and after a breastfeed and taking the increase in infant weight as the weight of milk consumed by the infant. Although there was considerable variation in breast milk intakes during the first few days of life (Table 5), breast milk intake tended to increase from 21.5 ± 4.2 mL/day on day 1 to 495.3 ± 33.4 mL/day on day 7 to 673.6 ± 29 mL/day after 14 days. 

Formula intake is generally measured by weighing the bottle weight before and after a feed and taking into account any spillage during feeding. Few studies reported formula intake during the first week of life, and hence meta-analyses were performed only for days 1, 2, and after 14 days since there were three or more studies for each of these times. Infant formula intake increased from 170.5 ± 55.8 mL/day on day 1 to 265.0 ± 67.7 mL/day on day 2 and to 761.8 ± 18 mL/day after 14 days (Table 6) and was greater than the volume of breast milk intake on all days analyzed. 

### 3.3. Energy Content of Breast Milk

Of the 25 potentially eligible papers identified by our literature search, 22 met the entry criteria, and these had a total of 387, 155, and 1088 breast milk samples for colostrum, transitional milk, and mature milk, respectively. The energy content of colostrum (53.6 ± 2.5 kcal/100 mL), transitional milk (57.7 ± 4.2 kcal/100 mL), and mature milk (65.2 ± 1.1 kcal/100 mL) was less than that commonly found in formula (67 kcal/100 mL), shown in Table 7. 

### 3.4. Macronutrient Intake in Breast-Fed and Formula-Fed Infants

The average volume of infant formula consumed was substantially higher than the volume of breast milk on all days analyzed. Due to the greater volume of milk intake by formula-fed infants and higher energy content of formula, (67 kcal/100 mL), average energy intake on the first day of life was 114 kcal/day in formula-fed infants compared to 12 kcal/day in breast-fed infants (a 9.5-fold difference). From day 14 up to 6 weeks of life, average energy intake in formula-fed infants remained higher at 513 kcal/day compared to 440 kcal/day in the breast-fed infants (a 1.2-fold difference). In addition, the greater volume of formula intake compared to breast milk intake led to an average protein intake on the first day to be 2.4 g in formula-fed infants compared to only 0.5 g in breast-fed infants (a 4.8-fold difference). By day 14, protein intake in formula-fed infants (10.7 g/day) was still slightly higher than that of breast-fed infants (8.8 g/day) (a 1.2-fold difference). A similar pattern of increased intake of carbohydrate and fat in the formula-fed infants compared to breast-fed infants was also observed.

## 4. Discussion

To our knowledge, this is the first meta-analysis of the macronutrient content of human milk which includes milk from early lactation. We found that, depending on number of days from birth, in the first two weeks of life, formula-fed infants have a 1.2- to 9.5-fold greater energy intake and a 1.2- to 4.8-fold greater protein intake than those breastfed. This difference is due to the higher energy and protein content of formula and a greater volume of intake which may contribute to greater weight gain in formula-fed compared to breast-fed infants during early infancy. These data are therefore consistent with the hypothesis that formula-fed infants may be overfed early in infancy during a possible critical period of growth that may lead to programming of long-term obesity [6, 8–10], and have implications for the optimal composition of infant formulas.

Ideally, the energy content of formula should be equivalent to corresponding energy content of human milk at different stages of lactation. However, formula is designed to be appropriate for the first year of life and most commercially available products have an energy density of approximately 67 kcal/100 mL, far greater than the energy content of early breast milk. We found the metabolizable energy content of mature milk (65.2 kcal/100 mL) was also slightly lower than infant formula (67 kcal/100 mL) and within the range reported by Neville (60.1 kcal/100 mL to 77.6 kcal/100 mL) [82]. Similarly, in a review of 25 studies, Reilly et al. reported a mean ME of mature human milk as 63.9 kcal/100 mL [39], while a doubly labeled water study demonstrated that the energy content of breast milk at five and 11 weeks was 57.4 kcal/100 mL and 59.8 kcal/100 mL, respectively [79]. Therefore, our findings suggest that formula-fed infants could have a higher energy intake in the first six months of life and could partially explain greater weight gain in infants given formula compared to breast milk.

Along with the increase in energy density at all stages of lactation, the volume of intake of formula-fed infants was greater than those breast fed on all days analyzed and in particular in the first days of life. There are several potential explanations for this. First, it is likely that milk supply is limited in the first 24–48 hours postpartum which was confirmed by this analysis. Breast-fed infants receive approximately 25 to 100 mL per day in the first two days of life. Second, mothers of formula-fed infants may encourage finishing of the bottle even though appetite has been satisfied, hence a greater volume of intake. The capacity of the stomach of the newborn is very small in the first two days of life and increases in volume capacity after three to four days of life [83].

In support of this, in a pilot study designed to test the hypothesis that a higher nutrient intake in the first postnatal week may increase later obesity risk, infants fed a lower energy formula did not compensate by consuming more volume [18]. These results are similar to that reported by Fomon et al. who illustrated that between eight and 41 days of life the volume consumed was not different between a calorically dense (100 kcal/100 mL) or calorically dilute (54 kcal/mL) formula. This leads to a greater energy intake and rate of growth in the infants consuming a more concentrated formula [84]. It is evident that during the early weeks of life infants drink to volume, not to energy needs. 

After this critical period, infants appear to regulate their volume of intake better during ad libitum feedings to meet caloric needs for growth. For instance, at about 6 weeks of age, infants fed either a calorically dilute (54 kcal/100 mL) or calorically dense (100 kcal/100 mL) formula modified their volume of intake so that energy intake was similar to that of infants fed a conventional formula (67 kcal/100 mL) [85]. Other studies provide support for this hypothesis. Small for gestation infants fed either a standard formula (65 kcal/100 mL) or calorically dense (87 kcal/mL) formula had similar intakes by two months of age [86].

The ability of an older infant (after 41 days) to regulate intake based on growth needs was validated by the fact that the mean weight gain between the two groups (calorically dilute and dense) during the “caloric matching phase” was nearly identical, 24.6 ± 4.6 and 24.9 ± 5.3 g/day [84]. Interestingly, during the “early window” where infants consumed similar volumes, there was a significant difference in weight gain between infants fed calorically dilute or dense formula. Infants consuming a calorically dense formula had a mean weight gain 1.4-fold greater (29.8 ± 4.9 versus 41 ± 10.4 g/day, *P* < 0.01) than those consuming the dilute formula which may be explained by a greater gain in percentage fat. The overall weight gain between the two groups up to 112 days was significantly different (26.3 ± 3.8 versus 30.2 ± 5.8, *P* < 0.05) which appears to be due to the initial difference in the early feeding period window [84]. Therefore, infants in the first weeks of life do not seem to compensate for getting less energy dense milk by drinking more (i.e., during the early weeks of life they drink to volume, not to energy needs). Hence, making the energy content of formula closer to that of breast milk may help reduce rapid weight gain of formula-fed infants in the critical window in the first weeks after birth. 

In addition to differences in energy intake between breast-fed and formula-fed infants, protein intake varies considerably between the two groups. Protein concentration of human milk is highest during the initial, colostrum period because it contains large amounts of immunoglobulins and lactoferrin which gradually decline to relatively low levels in mature milk. However, despite this higher protein concentration, the greater volume of milk intake by formula-fed infants means that formula-fed infants have up to a 5-fold higher protein intake in the first two weeks of life compared to those breast fed, a difference which is likely to contribute to faster weight gain in formula-fed compared to breast-fed infants [87]. 

 This faster weight gain in formula-fed infants compared to breast-fed infants is already evident in the first week of life. In two separate studies, healthy term breast-fed infants lost a mean 6.4% to 6.6% of birth weight before starting to gain weight compared to formula fed infants who only lost 3.5% to 3.7% [15, 16]. Breast-fed infants may not regain their birth weight by eight days of life; however, formula-fed infants generally exceed birth weight at eight days of age by 50–100 g [88, 89]. 

Importantly, several observational studies have demonstrated that faster postnatal weight gain in infancy leads to increased childhood and adult overweight status [17, 90–93], hypertension risk [94], and insulin resistance [91]. This has been shown across several different population groups including those of European [14] and Asian [94] decent. The critical window for these programming effects is not known, but recent experimental evidence [95] now supports observational data [14] suggesting that weight gain in the first week of life may be particularly influential for increasing the later risk of obesity. In a small pilot study, infants randomly assigned to receive a lower nutrient formula, designed to mimic the intake of the breast-fed infant for the first seven postnatal days, had lower weight and sum of skin-fold thicknesses at age six and 18 months than those given standard formula [95]. Nutrition and growth during a critical window in the first week of life could therefore influence the long-term risk of obesity.

Limitations of this meta-analysis include the relative heterogeneity of the studies used, the small sample size of several studies, and limited data from the first few days of life. A lack of stable isotope studies to measure the energy content of early breast milk, which are difficult to perform in the first weeks of life, is another key limitation. The measurement of breast milk volume (test weighing of infants) may be less accurate than measurement of formula intake. Despite these limitations, a consistent pattern of lower macronutrient intake in breast-fed babies is supported by the data demonstrating a slower rate of weight gain in breast-fed compared to formula-fed infants.

Breastfeeding is clearly the most optimal nutrition for an infant and has major advantages for health. Although clearly it is not possible to replicate these benefits in infant formula, research should continue to strive to mimic, as close as possible, the composition and intake of the breastfed infant to provide similar health benefits to infants who cannot be breastfed. This could possibly be achieved either by reducing energy and/or protein content of formula; or by reducing the volume of intake. Volume of formula intake is more difficult to manipulate safely and could lead to hypernatremia, hypoglycemia, unintended weight loss, and undue stress on the caregiver. Infants fed formula consume larger volumes than those breast fed in the early postnatal period. Therefore, a formula designed to achieve a growth rate similar to that of the breast-fed infant would need a lower protein and energy content than that in breast milk, to compensate for the higher volume of intake.

Here we demonstrate that the energy and macronutrient content of infant formula, as well as the volume of formula intake, may be increased compared to human milk. This difference, particularly in the first weeks of life, could contribute to a faster weight gain. This provides an opportunity to modify the energy content of current infant formulas to more closely match the intake of the breast-fed infant, a strategy that could help in the long-term prevention of obesity, metabolic disease, and cardiovascular disease.

## Figures and Tables

**Table 1:** Checklist for reporting systemic reviews, using the meta-analysis of observational studies in epidemiology [17].

Background: definition of the problem	(i) Limited information on the macronutrient and energy content of human breast milk during the first weeks of life
	(ii) Hypothesized infant formula may be too energy dense for infants during the first weeks of life

Search strategy	(i) Searched Medline database from inception until early 2011
	(ii) Hand-search relevant journals and references from identified studies
	(iii) Contact authors if additional information was needed to complete meta-analysis above what was published*

Inclusion and exclusion criteria	(i) Study included if healthy, term, singleton infants reporting values during the first month of life were reported
	(ii) Breast milk and infant formula daily intake values analyzed were for either exclusively breast-fed or formula-fed infants, respectively
	(iii) Only studies which reported new data were included, and duplicate publications (e.g., in reviews) were excluded

Methods	(i) Only studies that used valid methods were included, but no formal assessment of quality of included studies was completed

Results	(i) Give results of individual studies (in tables) and group estimates (in tables and text)
	(ii) Essential details of methodology and sample for each study included (in tables)

Discussion/conclusion	(i) Give generalization of the conclusions
	(ii) Need for further research

*All data was based on published studies with the exception of one dataset on intake of formula-fed infants [18].

**Table 2:** Lipid concentration in breast milk (g/100 mL).

Study	Colostrum 1–5 days (g/100 mL)	Transition 6–14 days (g/100 mL)	Mature >14 days* (g/100 mL)	*N*	Population
[19]			4.7 ± 0.4	13	USA
[20]	1.9 ± 0.4	2.9 ± 0.2	3.1 ± 0.2	10	Canada
[21]	1.0 ± 0.2	2.7 ± 0.4	4.3 ± 0.4	13,11,12	St. Lucia
[22]	2.5 ± 0.3		4.1 ± 0.2	10,29	Venezuela
[23]		2.8 ± 0.2		69	Italy
[24]			4.9 ± 0.3	13	USA
[25]			4.0 ± 0.3	12	USA
[26]	1.7 ± 0.2	3.5 ± 0.4	3.9 ± 0.5	10,12,12	USA
[27]	3.0 ± 0.1		3.6 ± 0.2	192,14	Japan
[28]	1.8 ± 0.3	3.8 ± 0.2	3.9 ± 0.2	18,22,23	USA
[29]			4.1 ± 0.1	71	Australia
[30]	2.5 ± 0.1		3.5 ± 0.2	65,64	Peru
[31]			4.0 ± 0.1	34	Australia
[32]	2.0 ± 0.1	2.5 ± 0.1	2.8 ± 0.1	41	India
[33]	2.6 ± 0.4		3.3 ± 0.1	7,24	Switzerland
[34]		3.1 ± 0.1	3.5 ± 0.1	48,46	Finland
[35]			3.6 ± 0.2	52	Italy
[36]	2.5 ± 0.7	3.3 ± 0.4	3.2 ± 0.6	3,5, 5	Netherlands
[37]			4.0 ± 0.0	52	North China
[38]	2.7 ± 0.2	2.8 ± 0.2	3.9 ± 0.2	21,39,40	Japan
Meta-analysis results	2.2 ± 0.2	3.0 ± 0.1	3.8 ± 0.1		

Reported as mean ± SEM.

*Mature milk > 14 days and <6 weeks.

**Table 3:** Carbohydrate concentration in breast milk (g/100 mL).

Study	Colostrum 1–5 days (g/100 mL)	Transition 6–14 days (g/100 mL)	Mature >14 days* (g/100 mL)	*N*	Population
[19]			6.4 ± 0.0	13	USA
[20]	5.1 ± 0.2	5.9 ± 0.2	6.5 ± 0.2	10	Canada
[41]	2.6 ± 0.0	4.1 ± 0.0		9	Australia
[22]	7.6 ± 0.3		8.3 ± 0.2	10,29	Venezuela
[42]	6.4 ± 0.2			19	USA
[43]	5.6 ± 0.1	6.3 ± 0.1	6.4 ± 0.1	46	Italy
[24]			7.1 ± 0.2	13	USA
[25]			6.3 ± 0.2	12	USA
[26]	6.1 ± 0.1	6.8 ± 0.2	7.1 ± 0.2	10,13,12	USA
[44]		5.5 ± 0.1	6.5 ± 0.1	77,47	Australia
[45]		5.9 ± 0.2	7.0 ± 0.2	15,11	Sweden
[30]	5.1 ± 0.9		6.4 ± 0.4	65,63	Peru
[31]			6.0 ± 0.1	18	Australia
[46]			7.3 ± 0.2	7	USA
[32]	5.2 ± 0.1	4.6 ± 0.1	5.0 ± 0.2	41	India
[47]	5.8 ± 0.1	6.7 ± 0.1	6.9 ± 0.1	24,24,22	USA
[34]		7.5 ± 0.1	7.7 ± 0.1	48,46	Finland
[35]			7.6 ± 0.2	41	North China
[36]	5.8 ± 0.4	5.8 ± 0.2	6.4 ± 0.3	3,5, 5	Netherlands
[48]	5.2 ± 0.1	6.2 ± 0.0		7,10	France
[38]	6.0 ± 0.1	6.0 ± 0.1	5.9 ± 0.1	21,39,40	Japan
Meta-analysis results	5.6 ± 0.6	5.9 ± 0.4	6.7 ± 0.2		

Reported as Mean ± SEM.

*Mature milk > 14 days and <6 weeks.

**Table 4:** Protein concentration in breast milk (g/100 mL).

Study	Colostrum 1–5 days (g/100 mL)	Transition 6–14 days (g/100 mL)	Mature >14 days* (g/100 mL)	*N*	Population
[19]			1.2 ± 0.1	13	USA
[22]	3.0 ± 0.2		1.2 ± 0.1	10,29	Venezuela
[52]	6.5 ± 0.7			15	Taiwan
[23]		1.9 ± 0.0		69	Italy
[53]	1.4 ± 0.1			16	Australia
[54]	3.8 ± 1.5	1.7 ± 0.1	0.9 ± 0.1	8,3, 6	USA
[24]			1.4 ± 0.1	13	USA
[26]	2.3 ± 0.1	1.6 ± 0.1	1.5 ± 0.1	12,13,12	USA
[27]	2.2 ± 0.1		1.4 ± 0.1	192,14	Japan
[45]		1.6 ± 0.1	0.9 ± 0.1	15	Sweden
[55]	2.4 ± 0.1	1.3 ± 0.1	0.8 ± 0.0	3,4, 7	USA
[30]	3.0 ± 0.2		1.6 ± 0.0	62,59	Peru
[31]			1.1 ± 0.0	18	Australia
[46]			1.8 ± 0.1	7	USA
[32]	1.9 ± 0.1	1.6 ± 0.1	1.3 ± 0.1	41	India
[47]	3.5 ± 0.2	2.5 ± 0.2	2.1 ± 0.2	22,24,21	USA
[34]		2.0 ± 0.0	1.5 ± 0.0	48,46	Finland
[56]	1.8 ± 0.1	1.4 ± 0.0	1.3 ± 0.1	28,47,28	Spain
[35]			1.2 ± 0.0	41	North China
[36]	3.1 ± 1.3	1.8 ± 0.0	1.4 ± 0.1	3,5, 5	Netherlands
[38]	1.8 ± 0.1	1.9 ± 0.1	1.7 ± 0.0	21,39,40	Japan
Meta-analysis results	2.5 ± 0.2	1.7 ± 0.1	1.3 ± 0.1		

Reported as mean ± SEM.

*Mature milk > 14 days and <6 weeks.

**Table 5:** Studies reporting infant breast milk intake.

Study	Method	Day 1 (mL/day)	Day 2 (mL/day)	Day 3 (mL/day)	Day 4 (mL/day)	Day 5 (mL/day)	Day 7 (mL/day)	>14 days* (mL/day)	*N*	Population
[19]	TW*							717 ± 37	13	USA
[20]	TW					384 ± 45		500 ± 59	10, 7	Canada
[57]	TW							728 ± 21	37	USA
[58]	TW							752 ± 38	20	USA
[59]	TW							710 ± 70	10	USA
[60]	TW	39 ± 14	120 ± 21	294 ± 43	420 ± 46	465 ± 26			11	USA
[42]	TW					539 ± 42		743 ± 44	19	USA
[61]	ISO**							891 ± 23	20	UK
[62]	TW							664 ± 38	15	Netherlands
[24]	TW							673 ± 53	13	USA
[63]	TW	31 ± 9	42 ± 10						15	Israel
[64]	TW	21 ± 5	88 ± 8	231 ± 13	371 ± 14	430 ± 16	452 ± 14		26 (d1) 88 (d2–7)	Australia
[65]	ISO							802 ± 57	10	UK
[66]	TW							582 ± 14	71	USA
[45]	TW						558 ± 21	724 ± 35	15, 11	Sweden
[67]	TW							758 ± 26	35	Denmark
[31]	TW							416 ± 24	34	Australia
[46]	TW							611 ± 41	7	USA
[68]	TW							818 ± 49	10	USA
[49]	TW	43 ± 28	177 ± 28	360 ± 47	437 ± 54	483 ± 38	556 ± 61	633 ± 50	6, 9, 10, 10, 11, 7, 9	USA
[47]	TW						441 ± 29	489 ± 35	22	USA
[69]	TW							600 ± 48	11	USA
[70]	TW	15 ± 1							90	Brazil
[71]	TW							671 ± 30	16	Netherlands
[72]	TW							671 ± 43	17	USA
Meta-analysis results		**21.5 ± 4.2**	**100.3 ± 21.8**	**285.3 ± 40.1**	**383.8 ± 17.4**	**457.2 ± 20.9**	**495.3 ± 33.4**	**673.6 ± 29.0**		

Reported as mean ± SEM.

*TW: test weighing method.

**IS: isotopic method.

**Table 6:** Studies reporting infant formula intake.

Study	Day 1** (mL/day)	Day 2** (mL/day)	Day 3 (mL/day)	Day 4 (mL/day)	Day 5 (mL/day)	Day 6 (mL/day)	Day 7 (mL/day)	>14 days* (mL/day)	*N*	Population
[59]								856.0 ± 41.4	10	USA
[73]								845.0 ± 26.9	18	USA
[62]								732.2 ± 29.5	12	Netherlands
[63]	61.1 ± 6.0	139.3 ± 8.9							28	Israel
[74]								755.0 ± 21.0 743.0 ± 21.0	45 42	USA, Canada
[75]	190.8 ± 8.3	297.4 ± 11.9	410 ± 11	495 ± 12	538 ± 13	560 ± 14	568 ± 14		d1–3 = 100, d4-5 = 99, d6 = 97, d7 = 89	UK
[76]								724.5 ± 28.0	12	USA
[68]								765.3 ± 42.3	10	USA
[18]	267.6 ± 28.9	361.9 ± 24.2	412 ± 26	493 ± 32	566 ± 30	555 ± 27	576 ± 29	696.8 ± 30.8	29	
Meta-analysis results	**170.5 ± 55.8**	**265.0 ± 67.7**						**761.8 ± 18**		

Reported as mean ± SEM.

All intakes were calculated using bottle weighing method.

*Mature milk > 14 days and <6 weeks.

**Meta-analysis was only done where there are at least 3 studies for given day.

**Table 7:** Metabolizable energy content of breast milk^1^.

Study	Colostrum 1–5 days (kcal/100 mL)	Transition 6–14 days (kcal/100 mL)	Mature >14 days* (kcal/100 mL)	Method	*N*	Population
[19]			72.7 ± 13.8	Calculated	13	USA
[20]	45.1 ± 16.5	56.5 ± 10.2		Calculated	10	Canada
[57]			63.4 ± 1.0**	Calculated	37	USA
[59]			63.2 ± 1.8**	Bomb calorimetry	10	USA
[22]	64.9 ± 4.4		74.9 ± 2.5	Calculated	10,29	Venezuela
[24]			78.2 ± 3.5	Calculated	13	USA
[25]			63.7 ± 2.6**	Bomb calorimetry	12	USA
[77]			67.1±1.7	Bomb calorimetry	10	USA
[26]	48.9 ± 9.2	65.1 ± 16.2	69.5±18.7	Calculated	10,12,12	USA
[27]	54.8 ± 0.5		61.6 ± 0.9**	Bomb calorimetry	227	Japan
[78]			61.9 ± 7.4**	Bomb calorimetry	11	Canada
[79]			61.3 ± 17.7**	Doubly labeled water	12	UK
[30]	54.9 ± 16.5		63.5 ± 16.5**	Calculated	65,63	Peru
[31]			64.0 ± 7.4**	Calculated	18	Australia
[32]	46.4 ± 1.5	47.3 ± 1.9	50.4 ± 2.0**	Calculated	41,41,41	India
[80]			68.5 ± 3.8	Calculated	25	USA
[34]		65.8 ± 1.8	67.8 ± 1.8	Calculated	48,46	Finland
[35]			67.6 ± 3.1	Calculated	41	North China
[36]	58.1 ± 13.2	60.1 ± 5.1	60.0 ± 6.7**	Calculated	3,5, 5	Netherlands
[81]			65.0 ± 0.5**	Calculated	415 (milk bank donors)	USA
[72]			65.4 ± 2.2**	Bomb calorimetry	8	USA
[38]	53.9 ± 2.8	56.8 ± 2.2	65.9 ± 2.6**	Calculated	21,39,40	Japan
Meta-analysis results	53.6 ± 2.5	57.7 ± 4.2	65.2 ± 1.1			

Reported as mean ± SEM.

^1^Gross energy content of breast milk was converted into metabolizable energy (ME) levels by multiplying the classic Atwater factors 4, 4, 9 kcal/g for carbohydrate, protein, and lipid, respectively, and by assuming 93% of gross breast milk energy reported by bomb calorimetry methods is metabolizable.

*Mature milk > 14 days and <6 weeks.

** Indicates value below current infant formula level 67.0** **kcal/100 mL.

## References

[1] Arenz S, Ruckerl R, Koletzko B (2004). Breast-feeding and childhood obesity—a systematic review. *International Journal of Obesity and Related Metabolic Disorders*.

[2] Harder T, Bergmann R, Kallischnigg G, Plagemann A (2005). Duration of breastfeeding and risk of overweight: a meta-analysis. *American Journal of Epidemiology*.

[3] Owen CG, Martin RM, Whincup PH, Davey-Smith G, Gillman MW, Cook DG (2005). The effect of breastfeeding on mean body mass index throughout life: a quantitative review of published and unpublished observational evidence. *American Journal of Clinical Nutrition*.

[4] Owen CG, Martin RM, Whincup PH, Smith GD, Cook DG (2005). Effect of infant feeding on the risk of obesity across the life course: a quantitative review of published evidence. *Pediatrics*.

[5] Singhal A, Cole TJ, Fewtrell M, Deanfield J, Lucas A (2004). Is slower early growth beneficial for long-term cardiovascular health?. *Circulation*.

[6] Singhal A, Lucas A (2004). Early origins of cardiovascular disease: is there a unifying hypothesis?. *The Lancet*.

[7] Singhal A, Wells J, Cole TJ, Fewtrell M, Lucas A (2003). Programming of lean body mass: a link between birth weight, obesity, and cardiovascular disease?. *American Journal of Clinical Nutrition*.

[8] Baird J, Fisher D, Lucas P, Kleijnen J, Roberts H, Law C (2005). Being big or growing fast: systematic review of size and growth in infancy and later obesity. *British Medical Journal*.

[9] Ong KKL, Preece MA, Emmett PM, Ahmed ML, Dunger DB (2002). Size at birth and early childhood growth in relation to maternal smoking, parity and infant breast-feeding: longitudinal birth cohort study and analysis. *Pediatric Research*.

[10] Monteiro POA, Victora CG (2005). Rapid growth in infancy and childhood and obesity in later life—a systematic review. *Obesity Reviews*.

[11] Durmus B, Mook-Kanamori DO, Holzhauer S (2010). Growth in foetal life and infancy is associated with abdominal adiposity at the age of 2 years: the generation R study. *Clinical Endocrinology*.

[12] Holzhauer S, Hokken Koelega ACS, Ridder MD (2009). Effect of birth weight and postnatal weight gain on body composition in early infancy. The Generation R Study. *Early Human Development*.

[13] Singhal A, Fewtrell M, Cole TJ, Lucas A (2003). Low nutrient intake and early growth for later insulin resistance in adolescents born preterm. *The Lancet*.

[14] Stettler N, Stallings VA, Troxel AB (2005). Weight gain in the first week of life and overweight in adulthood: a cohort study of European American subjects fed infant formula. *Circulation*.

[15] Crossland DS, Richmond S, Hudson M, Smith K, Abu-Harb M (2008). Weight change in the term baby in the first 2 weeks of life. *Acta Paediatrica*.

[16] Macdonald PD, Ross SRM, Grant L, Young D (2003). Neonatal weight loss in breast and formula fed infants. *Archives of Disease in Childhood*.

[17] Stroup DF, Berlin JA, Morton SC (2000). Meta-analysis of observational studies in epidemiology: a proposal for reporting. Meta-analysis of observational studies in epidemiology (MOOSE) group. *The Journal of the American Medical*.

[18] Singhal A Optigrow pilot study.

[19] Allen JC, Keller RP, Archer P, Neville MC (1991). Studies in human lactation: milk composition and daily secretion rates of macronutrients in the first year of lactation. *American Journal of Clinical Nutrition*.

[20] Anderson GH, Atkinson SA, Bryan MH (1981). Energy and macronutrient content of human milk during early lactation from mothers giving birth prematurely and at term. *American Journal of Clinical Nutrition*.

[21] Boersma ER, Offringa PJ, Muskiet FAJ, Chase WM, Simmons IJ (1991). Vitamin E, lipid fractions, and fatty acid composition of colostrum, transitional milk, and mature milk: an international comparative study. *American Journal of Clinical Nutrition*.

[22] Carias D, Velásquez G, Cioccia AM, Piñero D, Inciarte H, Hevia P (1997). The effect of lactation time on the macronutrient and mineral composition of milk from Venezuelan women. *Archivos Latinoamericanos de Nutricion*.

[23] Corvaglia L, Battistini B, Paoletti V, Aceti A, Capretti MG, Faldella G (2008). Near-infrared reflectance analysis to evaluate the nitrogen and fat content of human milk in neonatal intensive care units. *Archives of Disease in Childhood*.

[24] Dewey KG, Lonnerdal B (1983). Milk and nutrient intake of breast-fed infants from 1 to 6 months: relation to growth and fatness. *Journal of Pediatric Gastroenterology and Nutrition*.

[25] Ferris AM, Dotts MA, Clark RM, Ezrin M, Jensen RG (1988). Macronutrients in human milk at 2, 12, and 16 weeks postpartum. *Journal of the American Dietetic Association*.

[26] Gross SJ, David RJ, Bauman L, Tomarelli RM (1980). Nutritional composition of milk produced by mothers delivering preterm. *Journal of Pediatrics*.

[27] Hosoi S, Honma K, Daimatsu T, Kiyokawa M, Aikawa T, Watanabe S (2005). Lower energy content of human milk than calculated using conversion factors. *Pediatrics International*.

[28] Jackson MB, Lammi-Keefe CJ, Jensen RG, Couch SC, Ferris AM (1994). Total lipid and fatty acid composition of milk from women with and without insulin-dependent diabetes mellitus. *American Journal of Clinical Nutrition*.

[29] Kent JC, Mitoulas LR, Cregan MD, Ramsay DT, Doherty DA, Hartmann PE (2006). Volume and frequency of breastfeedings and fat content of breast milk throughout the day. *Pediatrics*.

[30] Marquis GS, Penny ME, Zimmer JP, Díaz JM, Marín RM (2003). An overlap of breastfeeding during late pregnancy is associated with subsequent changes in colostrum composition and morbidity rates among peruvian infants and their mothers. *Journal of Nutrition*.

[31] Mitoulas LR, Kent JC, Cox DB, Owens RA, Sherriff JL, Hartmann PE (2002). Variation in fat, lactose and protein in human milk over 24 h and throughout the first year of lactation. *British Journal of Nutrition*.

[32] Narang APS, Bains HS, Kansal S, Singh D (2006). Serial composition of human milk in preterm and term mothers. *Indian Journal of Clinical Biochemistry*.

[33] Ruegg M, Blanc B (1981). The fat globule size distribution in human milk. *Biochimica et Biophysica Acta*.

[34] Saarela T, Kokkonen J, Koivisto M (2005). Macronutrient and energy contents of human milk fractions during the first six months of lactation. *Acta Paediatrica*.

[35] Shehadeh N, Aslih N, Shihab S, Werman MJ, Sheinman R, Shamir R (2006). Human milk beyond one year post-partum: lower content of protein, calcium, and saturated very long-chain fatty acids. *Journal of Pediatrics*.

[36] Van Beusekom CM, Zeegers TA, Martini IA (1993). Milk of patients with tightly controlled insulin-dependent diabetes mellitus has normal macronutrient and fatty acid composition. *American Journal of Clinical Nutrition*.

[37] Wan ZX, Wang XL, Xu L, Geng Q, Zhang Y (2010). Lipid content and fatty acids composition of mature human milk in rural North China. *British Journal of Nutrition*.

[38] Yamawaki N, Yamada M, Kan-no T, Kojima T, Kaneko T, Yonekubo A (2005). Macronutrient, mineral and trace element composition of breast milk from Japanese women. *Journal of Trace Elements in Medicine and Biology*.

[39] Reilly JJ, Ashworth S, Wells JCK (2005). Metabolisable energy consumption in the exclusively breast-fed infant aged 3–6 months from the developed world: a systematic review. *British Journal of Nutrition*.

[40] Newburg DS, Neubauer SH, Jensen RG (1995). Carbohydrates in milk: analysis, quantities and significance. *Handbook of Milk Composition*.

[41] Arthur PG, Kent JC, Hartmann PE (1994). Metabolites of lactose synthesis in milk from diabetic and nondiabetic women during lactogenesis II. *Journal of Pediatric Gastroenterology and Nutrition*.

[42] Chen DC, Nommsen-Rivers L, Dewey KG, Lönnerdal B (1998). Stress during labor and delivery and early lactation performance. *American Journal of Clinical Nutrition*.

[43] Coppa GV, Gabrielli O, Pierani P, Catassi C, Carlucci A, Giorgi PL (1993). Changes in carbohydrate composition in human milk over 4 months of lactation. *Pediatrics*.

[44] Kulski JK, Hartmann PE (1981). Changes in human milk composition during the initiation of lactation. *Australian Journal of Experimental Biology & Medical Science*.

[45] Lonnerdal B, Forsum E, Hambraeus L (1976). A longitudinal study of the protein, nitrogen, and lactose contents of human milk from Swedish well nourished mothers. *American Journal of Clinical Nutrition*.

[46] Mohammad MA, Sunehag AL, Haymond MW (2009). Effect of dietary macronutrient composition under moderate hypocaloric intake on maternal adaptation during lactation. *American Journal of Clinical Nutrition*.

[47] Neubauer SH, Ferris AM, Chase CG (1993). Delayed lactogenesis in women with insulin-dependent diabetes mellitus. *American Journal of Clinical Nutrition*.

[48] Viverge D, Grimmonprez L, Cassanas G, Bardet L, Solere M (1990). Variations in oligosaccharides and lactose in human milk during the first week of lactation. *Journal of Pediatric Gastroenterology and Nutrition*.

[49] Neville MC, Keller R, Seacat J (1988). Studies in human lactation: milk volumes in lactating women during the onset of lactation and full lactation. *American Journal of Clinical Nutrition*.

[50] Bitman J, Carlson SE, Couch SC, Jensen RG (1995). Milk lipids. *Handbook of Milk Composition*.

[51] Atkinson SA, Lonnerdal B, Jensen RG (1995). Nitrogenous components of milk. *Handbook of Milk Composition*.

[52] Chao JCJ, Tseng HP, Chang CW (2004). Chicken extract affects colostrum protein compositions in lactating women. *Journal of Nutritional Biochemistry*.

[53] Cregan MD, De Mello TR, Kershaw D, McDougall K, Hartmann PE (2002). Initiation of lactation in women after preterm delivery. *Acta Obstetricia et Gynecologica Scandinavica*.

[54] Davis TA, Nguyen HV, Garcia-Bravo R (1994). Amino acid composition of human milk is not unique. *Journal of Nutrition*.

[55] Lonnerdal B, Woodhouse LR, Glazier C (1987). Compartmentalization and quantitation of protein in human milk. *Journal of Nutrition*.

[56] Sanchez-Pozo A, Lopez J, Pita ML (1986). Changes in the protein fractions of human milk during lactation. *Annals of Nutrition and Metabolism*.

[57] Butte NF, Garza C, O’Brian Smith E, Nichols BL (1984). Human milk intake and growth in exclusively breast-fed infants. *Journal of Pediatrics*.

[58] Butte NF, Wong WW, Klein PD, Garza C (1991). Measurement of milk intake: tracer-to-infant deuterium dilution method. *British Journal of Nutrition*.

[59] Butte NF, Wong WW, Ferlic L, O’Brian Smith E, Klein PD, Garza C (1990). Energy expenditure and deposition of breast-fed and formula-fed infants during early infancy. *Pediatric Research*.

[60] Casey CE, Neifert MR, Seacat JM, Neville MC (1986). Nutrient intake by breast-fed infants during the first five days after birth. *American Journal of Diseases of Children*.

[61] Davies PSW, Wells JCK, Lucas A (1994). Adjusting milk intake for body size in early infancy. *Early Human Development*.

[62] De Bruin NC, Degenhart HJ, Gàl S, Westerterp KR, Stijnen T, Visser HKA (1998). Energy utilization and growth in breast-fed and formula-fed infants measured prospectively during the first year of life. *American Journal of Clinical Nutrition*.

[63] Dollberg S, Lahav S, Mimouni FB (2001). A comparison of intakes of breast-fed and bottle-fed infants during the first two days of life. *Journal of the American College of Nutrition*.

[64] Evans KC, Evans RG, Royal R, Esterman AJ, James SL (2003). Effect of caesarean section on breast milk transfer to the normal term newborn over the first week of life. *Archives of Disease in Childhood*.

[65] Goldberg GR, Prentice AM, Coward WA (1991). Longitudinal assessment of the components of energy balance in well-nourished lactating women. *American Journal of Clinical Nutrition*.

[66] Krebs NF, Reidinger CJ, Robertson AD, Hambidge KM (1994). Growth and intakes of energy and zinc in infants fed human milk. *Journal of Pediatrics*.

[67] Michaelsen KF, Larsen PS, Thomsen BL, Samuelson G (1994). The Copenhagen Cohort Study on infant nutrition and growth: breast-milk intake, human milk macronutrient content, and influencing factors. *American Journal of Clinical Nutrition*.

[68] Motil KJ, Sheng HP, Montandon CM, Wong WW (1997). Human milk protein does not limit growth of breast-fed infants. *Journal of Pediatric Gastroenterology and Nutrition*.

[69] Pao EM, Himes JM, Roche AF (1980). Milk intakes and feeding patterns of breast-fed infants. *Journal of the American Dietetic Association*.

[70] Santoro W, Martinez FE, Ricco RG, Jorge SM (2010). Colostrum ingested during the first day of life by exclusively breastfed healthy newborn infants. *Journal of Pediatrics*.

[71] Van Raaij JMA, Schonk CM, Vermaat-Miedema SH, Peek MEM, Hautvast JGAJ (1991). Energy cost of lactation, and energy balances of well-nourished Dutch lactating women: reappraisal of the extra energy requirements of lactation. *American Journal of Clinical Nutrition*.

[72] Wood CS, Isaacs PC, Jensen M, Hilton HG (1988). Exclusively breast-fed infants: growth and caloric intake. *Pediatric nursing*.

[73] Butte NF, Wong WW, Garza C (1991). Energy requirements of breast-fed infants. *Journal of the American College of Nutrition*.

[74] Lloyd B, Halter RJ, Kuchan MJ, Baggs GE, Ryan AS, Masor ML (1999). Formula tolerance in postbreastfed and exclusively formula-fed infants. *Pediatrics*.

[75] James RJA, James A, Drewett RF, Cheetham TD (2007). Milk intake and feeding behavior in the first week of life and its relationship to cord blood ghrelin, leptin, and insulin concentrations. *Pediatric Research*.

[76] Montandon CM, Wills C, Garza C (1986). Formula intake of 1- and 4-month-old infants. *Journal of Pediatric Gastroenterology and Nutrition*.

[77] Garza C, Butte NF (1986). Energy concentration of human milk estimated from 24-h pools and various abbreviated sampling schemes. *Journal of Pediatric Gastroenterology and Nutrition*.

[78] Lepage G, Collet S, Bougle D (1984). The composition of preterm milk in relation to the degree of prematurity. *American Journal of Clinical Nutrition*.

[79] Lucas A, Ewing G, Roberts SB, Coward WA (1987). How much energy does the breast fed infant consume and expand?. *British Medical Journal*.

[80] Powe CE, Knott CD, Conklin-Brittain N (2010). Infant sex predicts breast milk energy content. *American Journal of Human Biology*.

[81] Wojcik KY, Rechtman DJ, Lee ML, Montoya A, Medo ET (2009). Macronutrient analysis of a nationwide sample of donor breast milk. *Journal of the American Dietetic Association*.

[82] Neville MC, Jensen RG (1995). Volume and caloric density of human milk. *Handbook of Milk Composition*.

[83] Zangen S, Di Lorenzo C, Zangen T, Mertz H, Schwankovsky L, Hyman PE (2001). Rapid maturation of gastric relaxation in newborn infants. *Pediatric Research*.

[84] Fomon SJ, Filer LJ, Thomas LN (1975). Influence of formula concentration on caloric intake and growth of normal infants. *Acta Paediatrica Scandinavica*.

[85] Fomon SJ (1974). Nutritional requirements in relation to growth. *Monatsschr Kinderheilkd*.

[86] Brooke OG, Kinsey JM (1985). High energy feeding in small for gestation infants. *Archives of Disease in Childhood*.

[87] Koletzko B, Von Kries R, Closa R (2009). Lower protein in infant formula is associated with lower weight up to age 2 y: a randomized clinical trial. *American Journal of Clinical Nutrition*.

[88] Fomon SJ, Thomas LN, Filer LJ, Ziegler EE, Leonard MT (1971). Food consumption and growth of normal infants fed milk-based formulas. *Acta Paediatrica Scandinavica, Supplement*.

[89] Ziegler EE (2006). Growth of breast-fed and formula-fed infants. *Nestle Nutrition Workshop Series: Paediatric Programme.*.

[90] Dubois L, Girard M (2006). Early determinants of overweight at 4.5 years in a population-based longitudinal study. *International Journal of Obesity*.

[91] Leunissen RWJ, Kerkhof GF, Stijnen T, Hokken-Koelega A (2009). Timing and tempo of first-year rapid growth in relation to cardiovascular and metabolic risk profile in early adulthood. *Journal of the American Medical Association*.

[92] Stettler N, Zemel BS, Kumanyika S, Stallings VA (2002). Infant weight gain and childhood overweight status in a multicenter, cohort study. *Pediatrics*.

[93] Taveras EM, Rifas-Shiman SL, Belfort MB, Kleinman KP, Oken E, Gillman MW (2009). Weight status in the first 6 months of life and obesity at 3 years of age. *Pediatrics*.

[94] Bowers K, Liu G, Wang P (2011). Birth weight, postnatal weight change, and risk for high blood pressure among Chinese children. *Pediatrics*.

[95] Singhal A, Lanigan J, Mackey A (2010). *Early Nutrition and Later Risk of Obesity*.

